# FOXS1 Promotes Tumor Progression by Upregulating CXCL8 in Colorectal Cancer

**DOI:** 10.3389/fonc.2022.894043

**Published:** 2022-07-08

**Authors:** Junfeng Qiu, Mingzhou Li, Cailin Su, Yihao Liang, Ruizhang Ou, Xiaoning Chen, Chengmei Huang, Yaxin Zhang, Yaping Ye, Wenting Liao, Chao Zhang

**Affiliations:** ^1^ Department of Pathology, Nanfang Hospital, Southern Medical University, Guangzhou, China; ^2^ Department of Pathology, School of Basic Medical Sciences, Southern Medical University, Guangzhou, China; ^3^ Department of Pathology, Guangdong Provincial Key Laboratory of Molecular Tumor Pathology, Guangzhou, China; ^4^ The Second Clinical College of Guangzhou University of Chinese Medicine, Guangzhou, China; ^5^ State Key Laboratory of Oncology in South China, Collaborative Innovation Center for Cancer Medicine, Sun Yat-Sen University Cancer Center, Guangzhou, China

**Keywords:** colorectal cancer, FOXS1, CXCL8, angiogenesis, invasion, metastasis

## Abstract

**Background:**

Forkhead box S1 (FOXS1) is a member of the forkhead box (FOX) transcriptional factor superfamily. The biological roles and underlying regulatory mechanism of FOXS1 in CRC remain unclear.

**Methods:**

Bioinformatics analysis, Western blotting, real-time PCR, and immunohistochemistry (IHC) were used to detect the expression FOXS1 in CRC. MTT assay, transwell assay, human umbilical vein endothelial cell tube formation assay, and chicken chorioallantoic membrane assay were performed to investigate the effects of FOXS1 on proliferation, invasion, and angiogenesis. Additionally, tumor formation assay and orthotopic implantation assay were used to investigate the effects of FOXS1 on tumor growth and metastasis *in vivo*. Furthermore, gene set enrichment analysis (GSEA) was used to analyze the correlation between FOXS1 and EMT or angiogenesis. The correlation between FOXS1 and CXCL8 expression was analyzed in clinical CRC samples using IHC.

**Results:**

The results showed that FOXS1 expression was upregulated in CRC tissues compared with adjacent normal intestine tissues. A high FOXS1 expression is positively correlated with poor survival. FOXS1 promoted the malignant behavior of CRC cancer cells *in vitro*, including proliferation, invasion, and angiogenesis. In addition, FOXS1 promoted tumor growth and metastasis in nude mice. Mechanistically, FOXS1 upregulated the expression of C–X–C motif chemokine ligand 8 (CXCL8) at the transcriptional level. Knockdown of CXCL8 blocked FOXS1 induced the enhancement of the EMT and angiogenesis. GSEAs in public CRC datasets revealed strong correlations between FOXS1 expression and EMT marker and angiogenesis markers. IHC showed that FOXS1 expression was positively correlated with CXCL8 expression and CD31 expression in clinical CRC samples.

**Conclusion:**

The results suggest that FOXS1 promotes angiogenesis and metastasis by upregulating CXCL8 in CRC. Interference with the FOXS1/CXCL8 axis may serve as a potential therapeutic target for the treatment of metastatic CRC.

## Introduction

Colorectal cancer (CRC) is the third most prevalent cancer in the world (1). About 20% of CRC patients have metastatic diseases in distant organs at the time of initial diagnosis (2). Despite of the improvement in combinational chemotherapy and targeted therapies, the prognosis of metastatic CRC remains poor. The 5-year survival rate of CRC patients with liver metastasis is only about 14% (3). Angiogenesis plays a critical role in tumor growth and metastatic spread in CRC. Targeting angiogenesis through the vascular endothelial growth factor (VEGF) pathway has become an essential part of current therapies against metastatic CRC (4). A total of four anti-angiogenetic drugs, namely, bevacizumab, aflibercept, ramucirumab, and regorafenib, have been approved by the US Food and Drug Administration for the treatment of metastatic CRC (5). However, the obtained survival benefit from anti-angiogenic drugs is limited due to a lack of validated predictive biomarkers and complicated resistance mechanisms (4). Therefore, development of valuable biomarkers and effective combinational therapeutic targets are urgently needed to impede the limitations of anti-angiogenic treatment for metastatic CRC patients.

Forkhead box S1 (FOXS1) is a member of the forkhead box (FOX) transcriptional factor superfamily. The FOX superfamily consists of 19 subclasses of Fox genes (FoxA to FoxS) (6). There are more than 50 FOX genes in the human genome. Each FOX gene contains a highly conserved forkhead (FKH) DNA-binding domain (7). Despite the similarity in their FKH DNA-binding domains, FOX proteins have divergent features and functions (8). For instance, the FOXP subfamily member FOXP3 is constitutively expressed in natural CD4^+^CD25^+^ T cells and has been shown to play critical roles during the differentiation and function of Treg cells (9). Unlike FOXP3, the FOXO subfamily members are mainly involved in the regulation of the cell cycle and are pivotal for the maintenance of tissue homeostasis in organs (10). Dysregulated FOX transcription factors are often found to be involved in developmental disorders and diseases such as cancer (11, 12). For example, FOXC1 and FOXC2 play critical roles in the development and metastasis of CRC (13, 14). FOXM1 is highly expressed in several cancers and has also been implicated in promoting angiogenesis, invasion, stemness, and drug resistance (15). Among FOX genes, FOXS1 is most closely homologous to FOXC1, FOXC2, and FOXL1 (8, 16). FOXS1 is predominantly expressed in the aorta and kidney (16). It is an early sensory neuronal marker (17). Early studies demonstrated that FOXS1 played critical roles in normal brain and testis development. FOXS1 deficiency leads to impaired sensory nerve cell development during embryonic development in mice and also results in a significant leakage of testicular blood vessels in mice (17–19). However, the expression pattern and biological function of FOXS1 in cancer are largely unknown.

In the current study, we reported that FOXS1 is upregulated in CRC. The overexpression of FOXS1 promotes tumor angiogenesis, invasion, and metastasis by upregulating C–X–C motif chemokine ligand 8 (CXCL8). These results uncover a novel role and underlying mechanism of FOXS1 in the progression of CRC, suggesting that the FOXS1/CXCL8 axis may be a promising anti-angiogenesis and tumor invasion target for the treatment of metastatic CRC.

## Materials and Methods

### Patients and Specimens

Eleven pairs of fresh biopsies (including CRC tumor tissues and their matched non-cancerous mucosa tissues) were obtained from the operation room of Nanfang Hospital, Southern Medical University. The fresh biopsies were stored in liquid nitrogen before usage. The tissue microarray containing 39 cases of CRC tissues were collected from Nanfang Hospital, Southern Medical University. The use of clinical materials for research purposes was approved by the Southern Medical University Institutional Board (Guangzhou, China), and informed consent of the patients was obtained before collection, following relevant regulations. All the specimens were pathologically diagnosed as colon cancer by the pathology department, and all the specimens of the patients had not received radiotherapy and chemotherapy.

### Cell Cultures

The human CRC cell lines HCT116, SW480, SW620, HT29, CaCo2, Colo205, DLD1, LS174T, HCT15, HCT8, RKO, SW837, and LoVo were purchased from the American Type Culture Collection and were stored in the Department of Pathology, Southern Medical University, China. SW620, SW480, and SW837 cells were cultured in Leibovitz’s L-15 medium (Gibco, Grand Island, NY, USA) supplemented with 10% fetal bovine serum (FBS) (Gibco). HCT116 cells were cultured in McCoy’s 5A medium (Gibco) with 10% FBS. LoVo, Caco2, and HT29 cells were cultured in DMEM medium (Gibco) supplemented with 10% FBS. HCT15, DLD1, LS174T, HCT8, RKO, and Colo205 cells were cultured in RPMI 1640 medium (Gibco) with 10% FBS. Human umbilical vein endothelial cells (HUVECs) were cultured in DMEM containing F12K medium (Gibco) and 20% FBS (Gibco). All cells were cultured at 37°C in a 5% CO_2_ atmosphere.

### Plasmids

The FOXS1 construct was generated by cloning PCR-amplified full-length human FOXS1 cDNA into pLenti-EF1a. To delete FOXS1, two short hairpin RNA (shRNA) sequences (FOXS1 shRNA#1: CCGGTAGTGGCATCTACCGCTACACTCGAGAGTGGCATCTACCGCTACATTTTTG;FOXS1shRNA#2:CCGGTCACTCAACGAGTGCTTTGTCTCGAGCACTCAACGAGTGCTTTGTTTTTTG) were cloned into pLKO.1. Vectors pLenti-EF1a and pLKO.1 were purchased from Addgene Inc. (Watertown, MA, USA).

### RNA Isolation, Reverse Transcription, and Real-Time PCR

RNA was isolated by RNAiso Plus (Takara, 9109). Real-time quantitative reverse transcription PCR (real-time qPCR) was performed in triplicate using SYBR Green Mix (Vazyme, Nanjing, China, Q711-02) and the ABI PRISM 7500 Sequence Detection System (Applied Biosystems, Foster City, CA, USA). The multiple-ratio relationship of the target gene expression between the experimental group and control group was calculated using the 2^-ΔΔCT^ method. The housekeeping gene GAPDH was used as an internal control. Primer Express was used to design the real-time PCR primers, and primer sequences for amplification are shown in **Supplementary Table S1**.

### Western Blotting Analysis

Western blotting analysis was performed as previously described (20), using the FOXS1 (Sigma, St. Louis, MO, USA, HPA042475), anti-CXCL8 (Bioss, Woburn, MA, USA, bs-0780R), and anti-Flag (Bioss, bs-10583R) antibodies. Mouse monoclonal anti-α-tubulin antibody (Sigma, #9026) was used as the internal control.

### Immunohistochemistry

Immunohistochemistry (IHC) staining and scoring were performed as previously described (21) using anti-FOXS1 (Sigma, HPA042475), anti-CD31 (Abcam, Cambridge, MA, USA, ab9498), and anti-CXCL8 (Bioss, bs-0780R) antibodies. The degree of IHC staining was reviewed and scored independently by two observers. The intensity of staining was graded according to the following criteria: 0 (no staining), 1 (weak staining = light yellow), 2 (moderate staining = yellow brown), and 3 (strong staining = brown). The intensity of staining ≥2 was used to define tumors as high expression and ≤1 as low expression.

### Methyl Thiazolyl Tetrazolium Assay

Cells (1 × 10^3^) were seeded on 96-well plates and cultured for 24 h. Twenty microliters of 5 mg/ml methyl thiazolyl tetrazolium (MTT) (Sigma, St. Louis, MO, USA) was added to each well and incubated for 4 h. The reaction liquid was then removed, and 100 µl dimethyl sulfoxide (DMSO, Sigma, St. Louis, MO, USA) was added to the wells. The absorbance was measured at 570 nm with a Microplate Autoreader (Bio-Rad, Hercules, CA, USA). The experiment was repeated three times.

### Colony-Formation Assay

Tumor cells were seeded on a six-well plate (200 cells/well) and incubated at 37°C in a humidified 5% CO_2_ atmosphere incubator for 2 weeks. Cells were fixed and stained with crystal violet (KeyGEN BioTECH). Colonies containing 450 cells were counted. Three independent experiments were performed for each cell line.

### Migration Assay and Invasion Assay

Eight-micrometer-pore filter membranes of the Boyden chambers were used for the migration assay. Tumor cells (1 × 10^5^) in culture medium containing 1% FBS were seeded in the upper chamber, and culture medium with 10% FBS was added in the lower chamber as a chemoattractant. For invasion assay, the upper side of the filter was coated with 0.2% Matrigel (BD Biosciences, San Jose, CA, USA). After incubation for 24 h, cells on the upper side of the filter were removed with cotton swabs. Cells that migrated to the lower surface of the filter were fixed in 4% paraformaldehyde and stained with crystal violet (KeyGEN BioTECH). Three independent experiments were performed.

### Chicken Chorioallantoic Membrane Assay

The chicken chorioallantoic membrane (CAM) assay was performed on the sixth day of development of fertilized chicken eggs as previously described (22).

### Human Umbilical Vein Endothelial Cell Tube Formation Assay

Precooled 24-well plate plates were coated with Matrigel Basement Membrane Matrix (BD Biosciences, Franklin Lakes, NJ, USA) and then allowed to polymerize at 37°C for 30 min. HUVECs (1 × 10^5^) in 200 ml of conditioned medium were added to each well and incubated at 37°C in 5% CO_2_ for 8 h. Images were obtained under a bright-field microscope, and the capillary tubes were quantified by counting the length. Three independent experiments were performed.

### Subcutaneous Tumor Formation and Orthotopic Mouse Metastatic Model

The female BALB/c nude mice (4 to 6 weeks old) were obtained from the Animal Center of Southern Medical University, Guangzhou, China. All mice with animal feeding certificates were raised in an SPF-level environment. All animal experiments were conducted in accordance with standard procedures and approved by the Institutional Use Committee for Animal Care. For subcutaneous tumor formation, 1 × 10^6^ cells were subcutaneously injected into the right dorsal flank of female BALB/c athymic nude mice (4–6 weeks of age, 18–20 g). Tumor volume (V) was monitored every 2 days by measuring the short axis (W) and long axis (L) of the xenograft tumor and calculated with the following formula: V = (L × W2)/2. All mice were sacrificed 19 days after inoculation. The tumors were excised and fixed with 10% neutral formalin and paraffin embedded for histology analysis. The orthotopic metastatic model was established as previously described (23). Nude mice were anesthetized and 1 × 10^6^ cells were injected into the cecal serosal layer. The mice were sacrificed when they reached endpoint criteria (moribund or lost 20% of their pre-experiment body weight). The individual organs were excised, and metastases were observed by histological analysis. The numbers of gross metastatic foci were determined using a dissection microscope.

### Statistical Analysis

SPSS 20.0 statistical software was used for data analysis. Two-tailed independent Student’s t-test was used to analyze two groups. The relationship between FOXS1 expression level and overall survival rate was calculated by the Kaplan–Meier method, and the difference in overall survival rate was statistically compared by the log-rank method. Spearman correlation analysis was used for expression correlation analysis. The relationship between pathological parameters was compared using the two-independent-sample non-parametric test and a chi-square test. One-way ANOVA test was used to compare the differences of data from multiple groups, and LSD-T was used for multiple comparisons. *p* < 0.05 was considered significant (* *p* < 0.05, ** *p* < 0.01, *** *p* < 0.001).

## Results

### FOXS1 Is Upregulated in CRC and Associated With a Poor Prognosis

**Figure 1:**
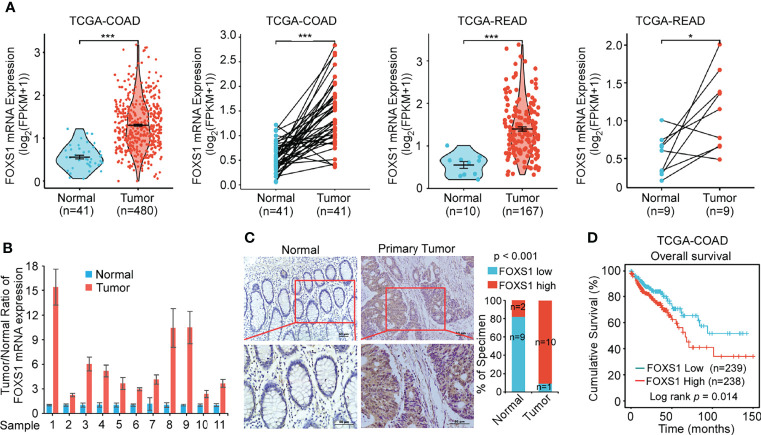
FOXS1 is upregulated in CRC and associated with a poor prognosis. **(A)** Analysis of FOXS1 expression in CRC compared with normal tissues in TCGA COAD and TCGA READ databases. FOXS1 expressions in paired CRC cancer tissues and adjacent normal tissues in TCGA COAD and TCGA READ datasets were also compared. **(B)** Real-time PCR analysis of FOXS1 expression in paired human CRC tissues and adjacent non-cancerous tissues. **(C)** The expression of FOXS1 in normal intestinal tissues and CRC tissues was examined by IHC. Representative IHC images (left) and correlation analysis (right) are shown. Scale bars: 50 µm. **(D)** Kaplan–Meier analysis of overall survival in all CRC patients in TCGA database according to FOXS1 expression (log-rank test, *p* = 0.014). *p < 0.05, ***p < 0.001.

The expression of FOXS1 expression was analyzed in TCGA COAD and READ databases, showing that FOXS1 was significantly upregulated in CRC tissues compared with that in normal colon and rectal tissues (**Figure 1A**). RT-PCR and IHC were used to detect the expression levels of FOXS1 in 11 CRC samples and their paired adjacent normal tissues. The results showed that the mRNA level of FOXS1 was significantly higher in CRC tissues than in the paired adjacent normal tissues (**Figure 1B**). IHC showed that positive FOXS1 expression was mainly localized in the nuclei and cytoplasm, as marked by yellow-brown staining (**Figure 1C**). Consistent with the mRNA expressions, IHC results showed that the protein level of FOXS1 was significantly higher in CRC tissues than in the paired adjacent normal tissues (**Figure 1C**). Kaplan–Meier survival analysis of TCGA-COAD datasets revealed a significantly poorer 5-year overall survival rate in patients with CRC with a high FOXS1 expression than in patients with a low FOXS1 expression (**Figure 1D**). These results suggested that FOXS1 is upregulated in CRC tissues.

### FOXS1 Promotes Proliferation and Invasiveness of CRC Cells

**Figure 2:**
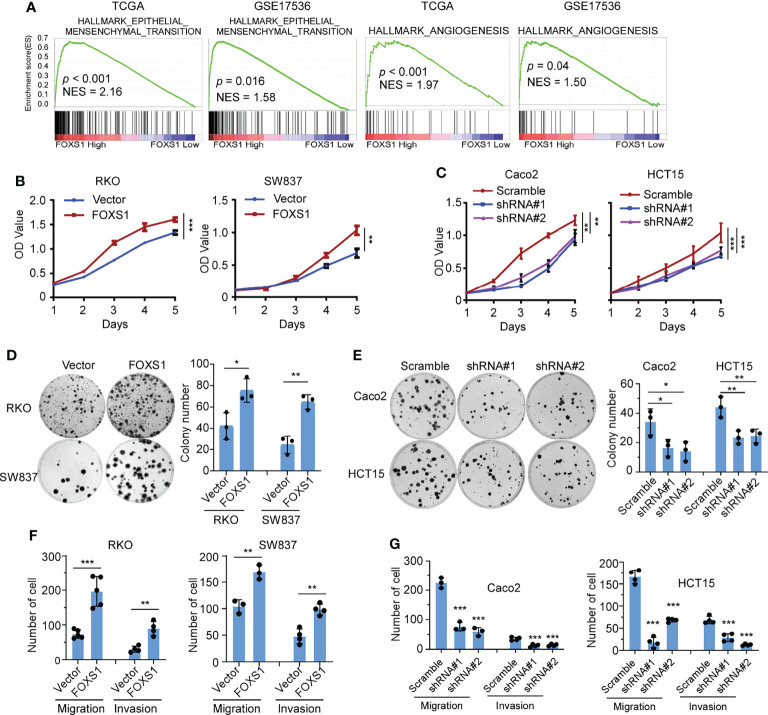
FOXS1 promotes proliferation and invasiveness of CRC cells *in vitro.* **(A)** GSEAs in TCGA-CRC and GSE17536 datasets. **(B, C)** MTT assays were performed to determine the effects of FOXS1 overexpression **(B)** or FOXS1 knockdown **(C)** on the proliferation of CRC cells. **(D, E)** Colony-formation assays were performed to determine the growth of FOXS1-overexpressed **(D)** or FOXS1-knockdown CRC cells **(E)**. **(F, G)** Transwell migration assay and invasion assay were performed to evaluate the migratory and invasive abilities of FOXS1-overexpressed **(F)** or FOXS1-knockdown CRC cells *in vitro* **(G)**. *p < 0.05, **p < 0.01, ***p < 0.001.

**Figure 3:**
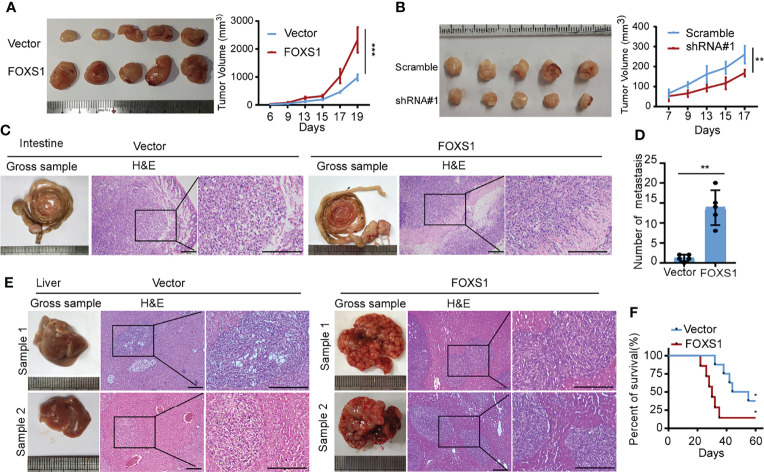
FOXS1 promotes tumor growth and metastasis *in vivo.***(A, B)** An *in vivo* tumorigenesis experiment was performed by subcutaneous injection in nude mice. The effect of FOXS1 overexpression on SW837 cells **(A)** or FOXS1 knockdown on HCT15 cells **(B)** was assessed by evaluating tumor volume (left) and tumor growth curves (right). **(C-F)** An orthotopic metastatic model was performed by injection of SW837 cells into the cecum. The mice were sacrificed when they reached the endpoint criteria (moribund or lost 20% of their pre-experiment body weight). **(C)** Representative gross images of the intestines are shown (left). Intestine sections were stained with hematoxylin and eosin (H&E, right). Scale bars: 100 µm. **(D)** The statistical analysis of the number of liver metastatic nodules. **(E)** Representative gross images of the livers and H&E staining for tissues are shown, and livers are shown (left). Intestines and liver sections were stained with hematoxylin and eosin (right). Scale bars: 100 µm. **(F)** The Kaplan–Meier method was used to analyze survival curves in the two groups, and the log-rank test was used to compare differences. **p < 0.01, ***p < 0.001.

A gene set enrichment analysis (GSEA) was used to examine the FOXS1-regulated gene signatures. According to the results of the GSEA, a higher FOXS1 expression was positively correlated with the enrichment of epithelial–mesenchymal transition and angiogenesis-related gene signatures (TCGA and GSE17536) (**Figure 2A**). To investigate the role of FOXS1 in CRC progression, stable overexpression cell lines were established in RKO and SW837 cell lines, which showed a low endogenous expression of FOXS1. In addition, endogenous FOXS1 was knocked down in CaCO_2_ and HCT15 CRC cells, which display a high endogenous FOXS1 expression (**Supplemental Figures S1A, B**). MTT assay and clone formation assay showed that FOXS1 overexpression significantly increased the proliferation, while knockdown of FOXS1 significantly decreased the cell proliferation *in vitro* (**Figures 2B–E**). Migration and invasion assays showed that overexpression of FOXS1 obviously increased the migratory and invasive abilities in RKO and SW837 cell lines (**Figure 2F** and **Supplemental Figure S2A**). On the contrary, knockdown of FOXS1 obviously suppressed the migratory and invasive abilities in CaCO_2_ and HCT15 cell lines (**Figure 2G** and **Supplemental Figure S2B**). Subcutaneous inoculation assay showed that overexpression of FOXS1 significantly increased (**Figure 3A**), while knockdown of FOXS1 significantly decreased the tumor growth in nude mice (**Figure 3B**). Orthotopic inoculation assay showed that overexpression of FOXS1 increased the liver metastases (**Figures 3C–E**) and shortened the overall survival of the mice (**Figure 3F**).

### FOXS1 Is Involved in Tumor Cell-Induced Angiogenesis

**Figure 4:**
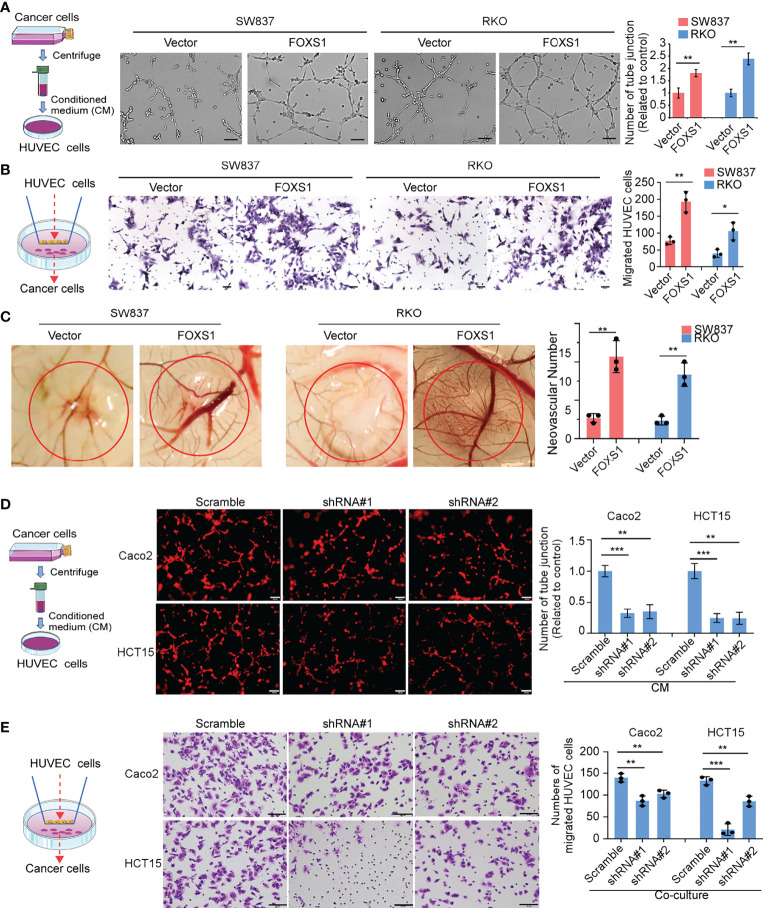
FOXS1 is involved in tumor cell-induced angiogenesis. **(A)** HUVEC tube formation after stimulation with the indicated conditional medium from vector control CRC cells and FOXS1-overexpressed CRC cells. Scale bars: 50 µm. The number of tube junctions was calculated (right). **(B)** Migration of HUVECs toward conditioned medium from vector control CRC cells and FOXS1-overexpressed CRC cells. Scale bars: 50 µm. The number of migrated HUVECs was calculated. **(C)** Representative images of the CAM assay. Histograms show the formation of secondary and tertiary blood vessels after stimulation with the indicated conditional medium from FOXS1-overexpressing cells. **(D)** HUVEC tube formation after stimulation with the indicated conditional medium from FOXS1-depleted CRC cells. Scale bar: 50 µm. The number of tube junctions was calculated (right). **(E)** Migration of HUVECs toward conditioned medium from FOXS1-depleted CRC cells. The number of migrated HUVECs was calculated. Scale bar: 50 µm. *p < 0.05, **p < 0.01, ***p < 0.001.

Since GSEAs revealed that a higher FOXS1 expression was positively correlated with the enrichment of the angiogenesis-related gene signature (**Figure 2A**), we performed tube formation, HUVEC migration assay, and chicken CAM assays to validate the effect of FOXS1 on tumor cell-induced angiogenesis. The results showed that overexpression of FOXS1 strongly promoted the formation of tubes by HUVECs (**Figure 4A**), promoted HUVEC migration toward the conditioned medium (**Figure 4B**), and increased angiogenesis in CAMs (**Figure 4C**). However, knockdown of FOXS1 significantly suppressed the formation of tubes by HUVECs (**Figure 4D**) and inhibited HUVEC migration toward the conditioned medium (**Figure 4E**). Moreover, FOXS1-overexpressing tumors had a higher microvascular density (indicated by CD31-positive cells) than control tumors (**Supplemental Figure S3A**). On the contrary, FOXS1-knockdown tumors had a decreased microvascular density (indicated by CD31-positive cells) as compared with control tumors (**Supplemental Figure S3B**). These results revealed that FOXS1 played an important role in tumor cell-induced angiogenesis in CRC.

### FOXS1 Promotes EMT and Angiogenesis by Upregulating CXCL8

**Figure 5:**
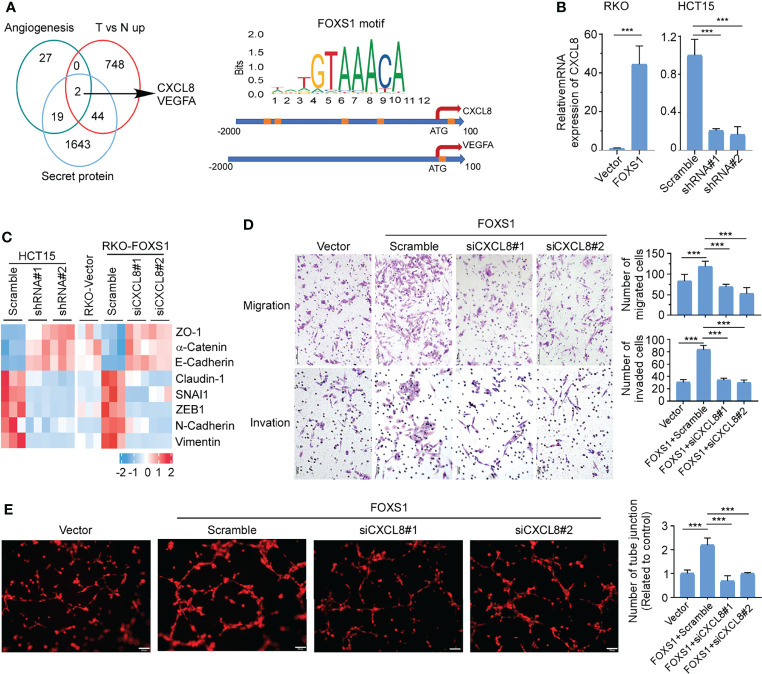
FOXS1 promotes EMT and angiogenesis by upregulating CXCL8. **(A)** Venn diagram analysis across three groups of genes (left). (i) Angiogenesis signature genes (n = 48); (ii) differentially expressed tumor versus normal in GSE18105 (n = 794); (iii) secreted protein genes (n = 1,708). The FOXS1 motif (orange boxes) was searched on the promoter loci of the CXCL8 gene and VEGFA gene in the JASPAR database. **(B)** Real-time PCR analysis of CXCL8 expression in CRC cell lines. **(C)** Real-time PCR analysis of the expression of epithelial–mesenchymal transition markers in CRC cell lines. **(D)** Transwell migration assay and transwell invasion assay were performed to determine the effects of CXCL8 depletion on the migratory and invasive abilities of FOXS1-overexpressed CRC cells. Scale bars: 50 µm. **(E)** HUVEC tube formation after stimulation with the indicated conditional medium from FOXS1-overexpressed CRC cells with or without CXCL8 depletion. Scale bar: 100 µm. ***p < 0.001.

Secreted proteins play integral roles in the cross talk between tumor cells and the tumor microenvironment. To identify essential genes that may underlie FOXS1-mediated angiogenesis and CRC development and progression, we integrated data across (i) angiogenesis signatures (**Supplementary Table S2**), (ii) genes that are upregulated in tumors versus normal tissues (GSE18105, **Supplementary Table S3**), and (iii) secretory proteins set from the Protein Atlas (https://www.proteinatlas.org/). These multidimensional datasets triangulated on two genes, CXCL8 and VEGFA (**Figure 5A**, left). To analyze whether FOXS1 would regulate the expression of CXCL8 and VEGFA at the transcriptional level, the FOXS1 motif (orange boxes) was searched on the promoter loci of the CXCL8 gene and VEGFA gene in the JASPAR database. Several FOXS1 motif loci were found on the CXCL8 promoter. Only one FOXS1 motif locus was found on the VEGFA promoter (**Figure 5A**, right). Q-RT-PCR showed that overexpression of FOXS1 significantly upregulated CXCL8, while knockdown of FOXS1 dramatically suppressed CXCL8 expression (**Figure 5B**). However, overexpression or knockdown of FOXS1 has no effect on the expression of VEGFA (**Supplemental Figure S4**). Next, we detected whether CXCL8 was involved in FOXS1-mediated EMT and angiogenesis. Q-RT-PCR (**Figure 5C**) showed that knockdown of FOXS1 significantly upregulated the expression of epithelial markers (ZO1, E-cadherin, and a-catenin), while it suppressed the mesenchymal markers (N-cadherin and vimentin) and EMT inducers (Claudin-1, SNAI1, and ZEB1). In contrast, overexpression of FOXS1 significantly suppressed the expression of ZO1, E-cadherin, and a-catenin, while it upregulated N-cadherin, vimentin, Claudin-1, SNAI1, and ZEB1. Knockdown of CXCL8 significantly downregulated N-cadherin, vimentin, Claudin-1, SNAI1, and ZEB1, while it upregulated ZO1, E-cadherin, and a-Catenin in FOXS1-overexpressing cells (**Figure 5C**). To investigate whether FOXS1 promotes EMT and angiogenesis by upregulating CXCL8, FOXS1-overexpressing cells were transfected with CXCL8 siRNAs. It was shown that knockdown of CXCL8 significantly suppressed the FOXS1-driven migration and invasiveness in CRC cells (**Figure 5D**) and inhibited the FOXS1-mediated tube formation ability *in vitro* (**Figure 5E**). These results suggested that FOXS1 promoted EMT and angiogenesis by upregulating CXCL8.

### FOXS1 Expression Positively Correlates With Angiogenesis and CXCL8 Expression in CRC

**Figure 6:**
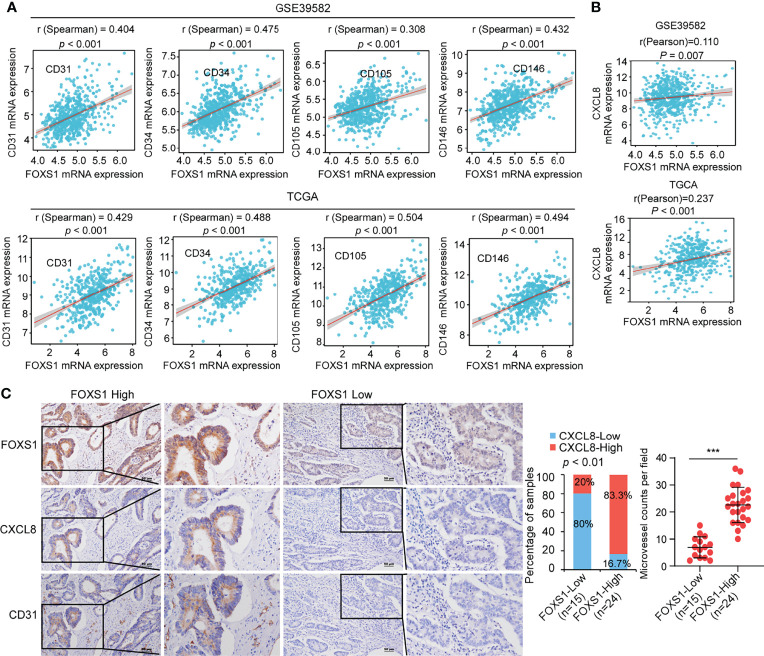
FOXS1 expression is positively correlated with angiogenesis and CXCL8 expression in CRC. **(A)** Correlation analysis of FOXS1 and CD31, CD34, CD105, or CD146 in GSE39582 and TCGA-CRC. **(B)** Correlation analysis of FOXS1 and CXCL8 in GSE39582 and TCGA-COAD (*r* = 0.110, *p* < 0.01; *r* = 0.237, *p* < 0.001). **(C)** The expressions of FOXS1, CXCL8, and CD31 protein in specimens, including 39 CRC tissue specimens, were examined by IHC. Representative IHC images (left) and correlation analysis (right) of FOXS1 and CXCL8 expression or microvessel count (indicated by CD31-positive cells) in per field are shown. Scale bars: 50 µm. ***p < 0.001.

To assess a potential link between FOXS1 and angiogenesis expression in human CRC, correlation analyses were performed in GSE39582 and TCGA datasets. The results revealed a strong positive correlation between high expression levels of FOXS1 and angiogenesis markers (CD31, CD34, CD105, and CD146) (**Figure 6A**). In addition, FOXS1 was positively corelated with EMT inducers (SNAI1, ZEB1, ZEB2) and mesenchymal marker vimentin, while it was negatively correlated with the epithelial marker CDH1 in both TCGA and GSE39582 datasets (**Supplemental Figure S5**). Moreover, FOXS1 expression is positively correlated with CXCL8 in both TCGA and GSE39582 datasets (**Figure 6B**). Furthermore, the analysis of a CRC tissue microarray including 39 CRC tissues revealed a significant positive correlation between FOXS1 expression and CXCL8 expression or microvascular density (indicated by CD31-positive cells) (**Figure 6C**).

## Discussion

FOXS1 is a member of the forkhead box family of transcription factors. The role of FOXS1 in the development and progression of human cancers is largely unknown. There are only a few studies that have reported that FOXS1 was involved in the regulation of cancer cell biology in gastric cancer (24, 25), hepatocellular carcinoma (26, 27), and glioma cells (28). Nevertheless, the role of FOXS1 in cancer remains controversial according to the results from these studies. For examples, Lu reported that FOXS1 is downregulated in gastric cancer, and overexpression of FOXS1 inhibits proliferation, metastasis, and EMT in gastric cancer cell lines (25). On the contrary, Wang’s study in a large sample size of clinical tissues showed that FOXS1 is upregulated in gastric cancer, and a high expression of FOXS1 significantly correlated with an aggressive phenotype and poor prognosis in patients with gastric cancer. In addition, they found that overexpression of FOXS1 promoted cell proliferation and EMT (24). Bévant et al. showed that FOXS1 could be epigenetically silenced in non-tumor tissues through DNA hypermethylation. A high expression of FOXS1 predicted a poor prognosis in liver cancer. In addition, overexpression of FOXS1 induced EMT markers and promoted cell migration and metastasis in liver cancer (26). On the contrary, Lei et al. reported that FOXS1 is downregulated hepatocellular carcinoma and overexpression of FOXS1 inhibited the proliferation and migration of hepatocellular carcinoma cells (27). These studies demonstrated conflicting results, probably due to using different cell lines. In gastric cancer, FOXS1 overexpression inhibits EMT in SNU-216 cells (25) but promotes EMT in SGC7901 and BGC823 cells (24). In liver cancer, Bévant et al. showed that FOXS1 evidently promoted EMT in PLC/PRF/5 (26), while Lei et al. demonstrated that FOXS1 suppressed EMT in Huh7 and Hep3B cell lines (27). These opposite results suggest that the function of FOXS1 could be dependent on specific cell-specific contexts associated with different genetic/epigenetic backgrounds of cancer subtypes. In the present study, we found that FOXS1 was upregulated in TCGA CRC datasets and clinical CRC samples. FOXS1 expression was significantly correlated with a poorer prognosis for patients with CRC. Overexpression of FOXS1 promotes cancer cell proliferation, invasiveness, angiogenesis, tumor growth, and metastasis. FOXS1 protein shares a high degree of similarity with FOXC2, which induces EMT and promotes metastasis (29). Taken together, regardless of previously contradictory reports in gastric cancer and hepatocellular carcinoma, our results indicate that FOXS1 might be a candidate oncogene in CRC.

Metastasis is a complex, multigene-involved, multistage process. EMT and angiogenesis are important features of tumors during invasion and metastasis (30). Members of the forkhead transcription factors, e.g., FOXC1 and FOXC2, are involved in angiogenesis and metastasis through various pathways (29, 31–33). As the structure of FOXS1 is highly similar with FOXC2, we propose that they may share similarities in their biological functions in human cancers. Recently, it has been reported that FOXS1 promoted EMT and was related to temozolomide resistance in glioma cells (28). In addition, FOXS1 was regulated by GLI1 and miR-125a-5p. Overexpression of FOXS1 promoted cell proliferation and EMT in gastric cancer (24). Moreover, FOXS1 induced by TGFβ promoted a classical model of EMT by activating the expression of EMT-transcription factors (SNAI1, and SNAI2) in hepatocellular carcinoma (26). As compared with the role of FOXS1 in EMT, the function of FOXS1 tumor angiogenesis is rarely known. FOXS1 was expressed in blood vessel-associated smooth muscle cells and pericytes in the central nervous system (18). Deletion of FOXS1 led to deficiency in the development of the testicular vasculature (19). These studies implicated a potential role of FOXS1 in angiogenesis in tumor. In the present study, we provided evidence that FOXS1 not only promoted EMT in CRC cells but also was involved in angiogenesis in human cancers.

Angiogenesis is crucial for the growth and metastasis of aggressive tumors (34). VEGF, angiopoietins, Notch, and integrins are key signaling pathways that regulate and orchestrate angiogenesis (35). Anti-angiogenesis by targeting the VEGF pathway using monoclonal antibodies (mAbs), small molecules, and tyrosine kinase inhibitors (TKIs) is an important part of controlling cancer progression (36). However, the obtained survival benefits from the current anti-angiogenetic drugs are limited due to multiple resistance mechanisms (37). CXC chemokines containing the ELR motif (ELR+) are well known by their ability to promote angiogenesis (38). CXCL8, an ELR+ CXC chemokine, also known as IL8, has a demonstrated angiogenic activity (39, 40). Like other ELR+ CXCs chemokines, CXCL8 binds to and acts on CXCR1 and CXCR2, especially CXCR2 (41). The proangiogenic activity of CXCL8 occurs predominantly following the interaction with CXCR2 (42). CXCR2, as the putative receptor for ELR+ CXC chemokine-induced angiogenic activity, is widely expressed in tumor cells and stroma cells such as endothelial cells and leukocytes (42–44). It was reported that the CXCL8–CXCR2 axis induces VEGF transcription and stimulates the autocrine activation of VEGFR2 in endothelial cells (45). In addition to promoting angiogenesis, the CXCL8/CXCR2 axis also exerts multiple effects on proliferation, invasion, and migration of tumor cells (41). The CXCL8/CXCR2 axis plays important roles in the initiation and development of various cancers including CRC, breast cancer, prostate cancer, and lung cancer (46–49). CXCL8 is upregulated in various cancers and are correlated with tumor stages and patient prognosis (50). The expression of CXCL8 is inducible under hypoxic conditions in several types of tumor cells, mainly through the cooperative activation of interferon, NF-κB, and activator protein-1 (AP-1) (51–53). In this study, we demonstrated that CXCL8 was upregulated by a forkhead transcriptional factor, FOXS1. The FOXS1/CXCL8 axis promoted tumor cell migration, invasion, angiogenesis, and metastasis in CRC, suggesting that FOXS1/CXCL8 may be a novel target in disrupting tumor-associated angiogenesis and in controlling advanced CRC.

## Conclusion

In conclusion, our data suggest that FOXS1 has an essential role in CRC angiogenesis and metastasis by regulating CXCL8. Interference with the FOXS1/CXCL8 axis may represent a new anti-angiogenic treatment option to prevent or reduce the metastasis of CRC.

## Data Availability Statement

The raw data supporting the conclusions of this article will be made available by the authors, without undue reservation.

## Ethics Statement

The studies involving human participants were reviewed and approved by The Institutional Committee of Southern Medical University and Nanfang Hospital. The patients/participants provided their written informed consent to participate in this study. The animal study was reviewed and approved by Committee on the Ethics of Animal Experiments of Southern Medical University. Written informed consent was obtained from the individual(s) for the publication of any potentially identifiable images or data included in this article.

## Author Contributions

The first authors, JQ, ML, and CS, provided the study concept and designed this study. JQ and ML carried out the experiments and analyzed the data. ML wrote the first draft. The corresponding author WL was predominantly engaged in organizing, designing, and writing the article. Other authors contributed equally to the paper’s conception, literature review, writing, and editing of the figures. Each author approved the final version of the manuscript.

## Funding

This work was supported by grants from the National Natural Science Foundation of China (No. 81872401, 81672886).

## Conflict of Interest

The authors declare that the research was conducted in the absence of any commercial or financial relationships that could be construed as a potential conflict of interest.

## Publisher’s Note

All claims expressed in this article are solely those of the authors and do not necessarily represent those of their affiliated organizations, or those of the publisher, the editors and the reviewers. Any product that may be evaluated in this article, or claim that may be made by its manufacturer, is not guaranteed or endorsed by the publisher.

## References

[1] Siegel RL Miller KD Fuchs HE Jemal A. Cancer Statistics, 2021. CA Cancer J Clin (2021) 71(1):7–33. doi: 10.3322/caac.21654 33433946

[2] Brenner H Kloor M Pox CP. Colorectal Cancer. Lancet (2014) 383(9927):1490–502. doi: 10.1016/S0140-6736(13)61649-9 24225001

[3] Siegel RL Miller KD Goding Sauer A Fedewa SA Butterly LF Anderson JC. Colorectal Cancer Statistics, 2020. CA Cancer J Clin (2020) 70(3):145–64. doi: 10.3322/caac.21601 32133645

[4] Apte RS Chen DS Ferrara N. VEGF in Signaling and Disease: Beyond Discovery and Development. Cell (2019) 176(6):1248–64. doi: 10.1016/j.cell.2019.01.021 PMC6410740 30849371

[5] Mody K Baldeo C Bekaii-Saab T. Antiangiogenic Therapy in Colorectal Cancer. Cancer J (2018) 24(4):165–70. doi: 10.1097/PPO.0000000000000328 30119079

[6] Jackson BC Carpenter C Nebert DW Vasiliou V. Update of Human and Mouse Forkhead Box (FOX) Gene Families. Hum Genomics (2010) 4(5):345–52. doi: 10.1186/1479-7364-4-5-345 PMC3500164 20650821

[7] Hannenhalli S Kaestner KH. The Evolution of Fox Genes and Their Role in Development and Disease. Nat Rev Genet (2009) 10(4):233–40. doi: 10.1038/nrg2523 PMC2733165 19274050

[8] Golson ML Kaestner KH. Fox Transcription Factors: From Development to Disease. Development (2016) 143(24):4558–70. doi: 10.1242/dev.112672 PMC5201025 27965437

[9] Hori S Nomura T Sakaguchi S. Control of Regulatory T Cell Development by the Transcription Factor Foxp3. Science (2003) 299(5609):1057–61. doi: 10.1126/science.1079490 12522256

[10] Calnan DR Brunet A. The FoxO Code. Oncogene (2008) 27(16):2276–88. doi: 10.1038/onc.2008.21 18391970

[11] Myatt SS Lam EW. The Emerging Roles of Forkhead Box (Fox) Proteins in Cancer. Nat Rev Cancer (2007) 7(11):847–59. doi: 10.1038/nrc2223 17943136

[12] Laissue P. The Forkhead-Box Family of Transcription Factors: Key Molecular Players in Colorectal Cancer Pathogenesis. Mol Cancer (2019) 18(1):5. doi: 10.1186/s12943-019-0938-x 30621735 PMC6325735

[13] Cui YM Jiang D Zhang SH Wu P Ye YP Chen CM. FOXC2 Promotes Colorectal Cancer Proliferation Through Inhibition of FOXO3a and Activation of MAPK and AKT Signaling Pathways. Cancer Lett (2014) 353(1):87–94. doi: 10.1016/j.canlet.2014.07.008 25069037

[14] Liu J Zhang Z Li X Chen J Wang G Tian Z. Forkhead Box C1 Promotes Colorectal Cancer Metastasis Through Transactivating ITGA7 and FGFR4 Expression. Oncogene (2018) 37(41):5477–91. doi: 10.1038/s41388-018-0355-4 29884889

[15] Kalathil D John S Nair AS. FOXM1 and Cancer: Faulty Cellular Signaling Derails Homeostasis. Front Oncol (2020) 10:626836. doi: 10.3389/fonc.2020.626836 33680951 PMC7927600

[16] Cederberg A Betz R Lagercrantz S Larsson C Hulander M Carlsson P. Chromosome Localization, Sequence Analysis, and Expression Pattern Identify FKHL 18 as a Novel Human Forkhead Gene. Genomics (1997) 44(3):344–6. doi: 10.1006/geno.1997.4864 9325056

[17] Montelius A Marmigere F Baudet C Aquino JB Enerback S Ernfors P. Emergence of the Sensory Nervous System as Defined by Foxs1 Expression. Differentiation (2007) 75(5):404–17. doi: 10.1111/j.1432-0436.2006.00154.x 17309606

[18] Heglind M Cederberg A Aquino J Lucas G Ernfors P Enerback S. Lack of the Central Nervous System- and Neural Crest-Expressed Forkhead Gene Foxs1 Affects Motor Function and Body Weight. Mol Cell Biol (2005) 25(13):5616–25. doi: 10.1128/MCB.25.13.5616-5625.2005 PMC1157007 15964817

[19] Sato Y Baba T Zubair M Miyabayashi K Toyama Y Maekawa M. Importance of Forkhead Transcription Factor Fkhl18 for Development of Testicular Vasculature. Mol Reprod Dev (2008) 75(9):1361–71. doi: 10.1002/mrd.20888 18288644

[20] Liao WT Jiang D Yuan J Cui YM Shi XW Chen CM. HOXB7 as a Prognostic Factor and Mediator of Colorectal Cancer Progression. Clin Cancer Res (2011) 17(11):3569–78. doi: 10.1158/1078-0432.CCR-10-2533 21474578

[21] Wang S Qiu J Liu L Su C Qi L Huang C. CREB5 Promotes Invasiveness and Metastasis in Colorectal Cancer by Directly Activating MET. J Exp Clin Cancer Res (2020) 39(1):168. doi: 10.1186/s13046-020-01673-0 32843066 PMC7446182

[22] Jiao HL Ye YP Yang RW Sun HY Wang SY Wang YX. Downregulation of SAFB Sustains the NF-kappaB Pathway by Targeting TAK1 During the Progression of Colorectal Cancer. Clin Cancer Res (2017) 23(22):7108–18. doi: 10.1158/1078-0432.CCR-17-0747 28912140

[23] Xu Y Zhang L Wang Q Zheng M. Comparison of Different Colorectal Cancer With Liver Metastases Models Using Six Colorectal Cancer Cell Lines. Pathol Oncol Res (2020) 26(4):2177–83. doi: 10.1007/s12253-020-00805-3 32172478

[24] Wang S Ran L Zhang W Leng X Wang K Liu G. FOXS1 is Regulated by GLI1 and miR-125a-5p and Promotes Cell Proliferation and EMT in Gastric Cancer. Sci Rep (2019) 9(1):5281. doi: 10.1038/s41598-019-41717-w 30918291 PMC6437149

[25] Lu Q Ma X Li Y Song W Zhang L Shu Y. Overexpression of FOXS1 in Gastric Cancer Cell Lines Inhibits Proliferation, Metastasis, and Epithelial-Mesenchymal Transition of Tumor Through Downregulating Wnt/Beta-Catenin Pathway. J Cell Biochem (2019) 120(3):2897–907. doi: 10.1002/jcb.26821 30500980

[26] Bevant K Desoteux M Angenard G Pineau R Caruso S Louis C. TGFbeta-Induced FOXS1 Controls Epithelial-Mesenchymal Transition and Predicts a Poor Prognosis in Liver Cancer. Hepatol Commun (2021) 6(5):1157–71.. doi: 10.1002/hep4.1866. PMC9035581 34825776

[27] Lei D Hu G Chen Y Hao T Gao Y Luo F. Forkhead Box S1 Inhibits the Progression of Hepatocellular Carcinoma. Onco Targets Ther (2020) 13:11839–48. doi: 10.2147/OTT.S272596 PMC7680191 33235470

[28] Xue BZ Xiang W Zhang Q Wang HF Zhou YJ Tian H. CD90(low) Glioma-Associated Mesenchymal Stromal/Stem Cells Promote Temozolomide Resistance by Activating FOXS1-Mediated Epithelial-Mesenchymal Transition in Glioma Cells. Stem Cell Res Ther (2021) 12(1):394. doi: 10.1186/s13287-021-02458-8 34256854 PMC8278613

[29] Hollier BG Tinnirello AA Werden SJ Evans KW Taube JH Sarkar TR. FOXC2 Expression Links Epithelial-Mesenchymal Transition and Stem Cell Properties in Breast Cancer. Cancer Res (2013) 73(6):1981–92. doi: 10.1158/0008-5472.CAN-12-2962 PMC3602160 23378344

[30] Nguyen DX Bos PD Massague J. Metastasis: From Dissemination to Organ-Specific Colonization. Nat Rev Cancer (2009) 9(4):274–84. doi: 10.1038/nrc2622 19308067

[31] Mani SA Yang J Brooks M Schwaninger G Zhou A Miura N. Mesenchyme Forkhead 1 (FOXC2) Plays a Key Role in Metastasis and is Associated With Aggressive Basal-Like Breast Cancers. Proc Natl Acad Sci U S A (2007) 104(24):10069–74. doi: 10.1073/pnas.0703900104 PMC1891217 17537911

[32] Hayashi H Sano H Seo S Kume T. The Foxc2 Transcription Factor Regulates Angiogenesis *via* Induction of Integrin Beta3 Expression. J Biol Chem (2008) 283(35):23791–800. doi: 10.1074/jbc.M800190200 PMC2527100 18579532

[33] Sizemore ST Keri RA. The Forkhead Box Transcription Factor FOXC1 Promotes Breast Cancer Invasion by Inducing Matrix Metalloprotease 7 (MMP7) Expression. J Biol Chem (2012) 287(29):24631–40. doi: 10.1074/jbc.M112.375865 PMC3397891 22645147

[34] Folkman J. Role of Angiogenesis in Tumor Growth and Metastasis. Semin Oncol (2002) 29(6 Suppl 16):15–8. doi: 10.1053/sonc.2002.37263 12516034

[35] Jain RK. Normalization of Tumor Vasculature: An Emerging Concept in Antiangiogenic Therapy. Science (2005) 307(5706):58–62. doi: 10.1126/science.1104819 15637262

[36] Folkman J. Angiogenesis: An Organizing Principle for Drug Discovery? Nat Rev Drug Discov (2007) 6(4):273–86. doi: 10.1038/nrd2115 17396134

[37] Jayson GC Kerbel R Ellis LM Harris AL. Antiangiogenic Therapy in Oncology: Current Status and Future Directions. Lancet (2016) 388(10043):518–29. doi: 10.1016/S0140-6736(15)01088-0 26853587

[38] Strieter RM Polverini PJ Kunkel SL Arenberg DA Burdick MD Kasper J. The Functional Role of the ELR Motif in CXC Chemokine-Mediated Angiogenesis. J Biol Chem (1995) 270(45):27348–57. doi: 10.1074/jbc.270.45.27348 7592998

[39] Heidemann J Ogawa H Dwinell MB Rafiee P Maaser C Gockel HR. Angiogenic Effects of Interleukin 8 (CXCL8) in Human Intestinal Microvascular Endothelial Cells are Mediated by CXCR2. J Biol Chem (2003) 278(10):8508–15. doi: 10.1074/jbc.M208231200 12496258

[40] Li A Dubey S Varney ML Dave BJ Singh RK. IL-8 Directly Enhanced Endothelial Cell Survival, Proliferation, and Matrix Metalloproteinases Production and Regulated Angiogenesis. J Immunol (2003) 170(6):3369–76. doi: 10.4049/jimmunol.170.6.3369 12626597

[41] Liu Q Li A Tian Y Wu JD Liu Y Li T. The CXCL8-CXCR1/2 Pathways in Cancer. Cytokine Growth Factor Rev (2016) 31:61–71. doi: 10.1016/j.cytogfr.2016.08.002 27578214 PMC6142815

[42] Addison CL Daniel TO Burdick MD Liu H Ehlert JE Xue YY. The CXC Chemokine Receptor 2, CXCR2, is the Putative Receptor for ELR+ CXC Chemokine-Induced Angiogenic Activity. J Immunol (2000) 165(9):5269–77. doi: 10.4049/jimmunol.165.9.5269 11046061

[43] Saintigny P Massarelli E Lin S Ahn YH Chen Y Goswami S. CXCR2 Expression in Tumor Cells is a Poor Prognostic Factor and Promotes Invasion and Metastasis in Lung Adenocarcinoma. Cancer Res (2013) 73(2):571–82. doi: 10.1158/0008-5472.CAN-12-0263 PMC3548940 23204236

[44] Liao W Overman MJ Boutin AT Shang X Zhao D Dey P. KRAS-IRF2 Axis Drives Immune Suppression and Immune Therapy Resistance in Colorectal Cancer. Cancer Cell (2019) 35(4):559–72.e7. doi: 10.1016/j.ccell.2019.02.008 30905761 PMC6467776

[45] Martin D Galisteo R Gutkind JS. CXCL8/IL8 Stimulates Vascular Endothelial Growth Factor (VEGF) Expression and the Autocrine Activation of VEGFR2 in Endothelial Cells by Activating NFkappaB Through the CBM (Carma3/Bcl10/Malt1) Complex. J Biol Chem (2009) 284(10):6038–42. doi: 10.1074/jbc.C800207200 PMC2649103 19112107

[46] Cheng XS Li YF Tan J Sun B Xiao YC Fang XB. CCL20 and CXCL8 Synergize to Promote Progression and Poor Survival Outcome in Patients With Colorectal Cancer by Collaborative Induction of the Epithelial-Mesenchymal Transition. Cancer Lett (2014) 348(1-2):77–87. doi: 10.1016/j.canlet.2014.03.008 24657657

[47] Arenberg DA Kunkel SL Polverini PJ Glass M Burdick MD Strieter RM. Inhibition of Interleukin-8 Reduces Tumorigenesis of Human non-Small Cell Lung Cancer in SCID Mice. J Clin Invest (1996) 97(12):2792–802. doi: 10.1172/JCI118734 PMC507372 8675690

[48] Lin Y Huang R Chen L Li S Shi Q Jordan C. Identification of Interleukin-8 as Estrogen Receptor-Regulated Factor Involved in Breast Cancer Invasion and Angiogenesis by Protein Arrays. Int J Cancer (2004) 109(4):507–15. doi: 10.1002/ijc.11724 14991571

[49] Inoue K Slaton JW Eve BY Kim SJ Perrotte P Balbay MD. Interleukin 8 Expression Regulates Tumorigenicity and Metastases in Androgen-Independent Prostate Cancer. Clin Cancer Res (2000) 6(5):2104–19. 10815938

[50] Ha H Debnath B Neamati N. Role of the CXCL8-CXCR1/2 Axis in Cancer and Inflammatory Diseases. Theranostics (2017) 7(6):1543–88. doi: 10.7150/thno.15625 PMC5436513 28529637

[51] Singh RK Varney ML. Regulation of Interleukin 8 Expression in Human Malignant Melanoma Cells. Cancer Res (1998) 58(7):1532–7. 9537260

[52] Kunz M Hartmann A Flory E Toksoy A Koczan D Thiesen HJ. Anoxia-Induced Up-Regulation of Interleukin-8 in Human Malignant Melanoma. A Potential Mechanism for High Tumor Aggressiveness. Am J Pathol (1999) 155(3):753–63. doi: 10.1016/S0002-9440(10)65174-7 PMC1866897 10487833

[53] Xu L Xie K Mukaida N Matsushima K Fidler IJ. Hypoxia-Induced Elevation in Interleukin-8 Expression by Human Ovarian Carcinoma Cells. Cancer Res (1999) 59(22):5822–9. 10582705

