# Supplementary figures and images for: FOXS1 Promotes Tumor Progression by Upregulating CXCL8 in Colorectal Cancer
Source: Front Oncol. 2022 Jul 8;12:894043. doi: 10.3389/fonc.2022.894043 (PMC9309265; doi:10.3389/fonc.2022.894043)

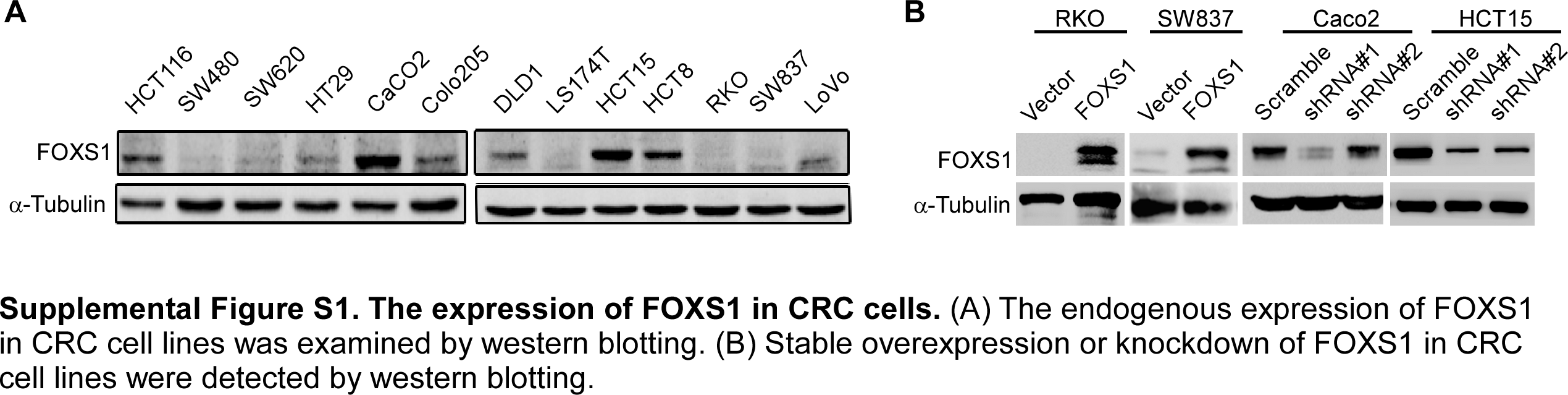

Supplement: Supplementary file 1 [file Image_1.tif]

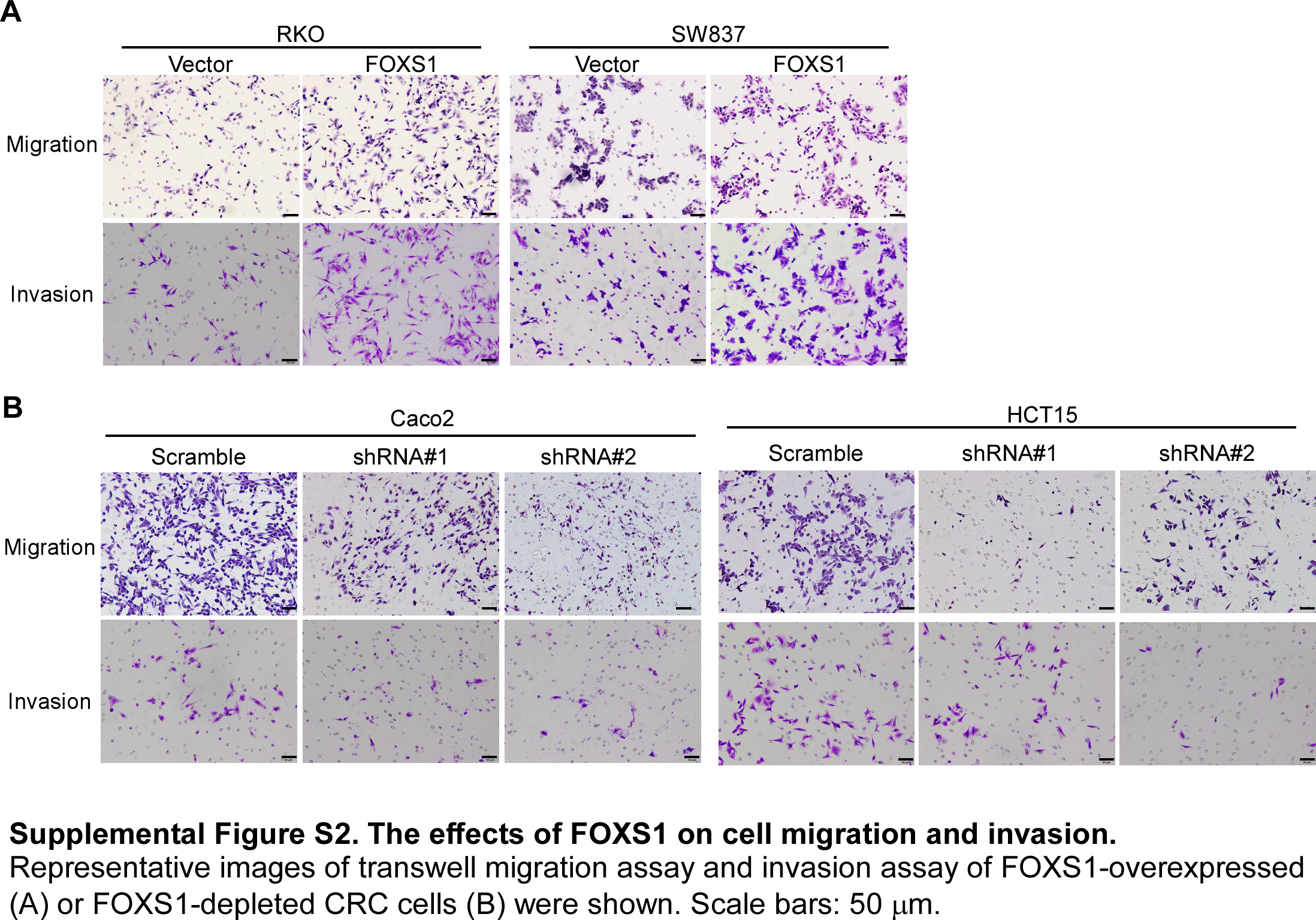

Supplement: Supplementary file 2 [file Image_2.tif]

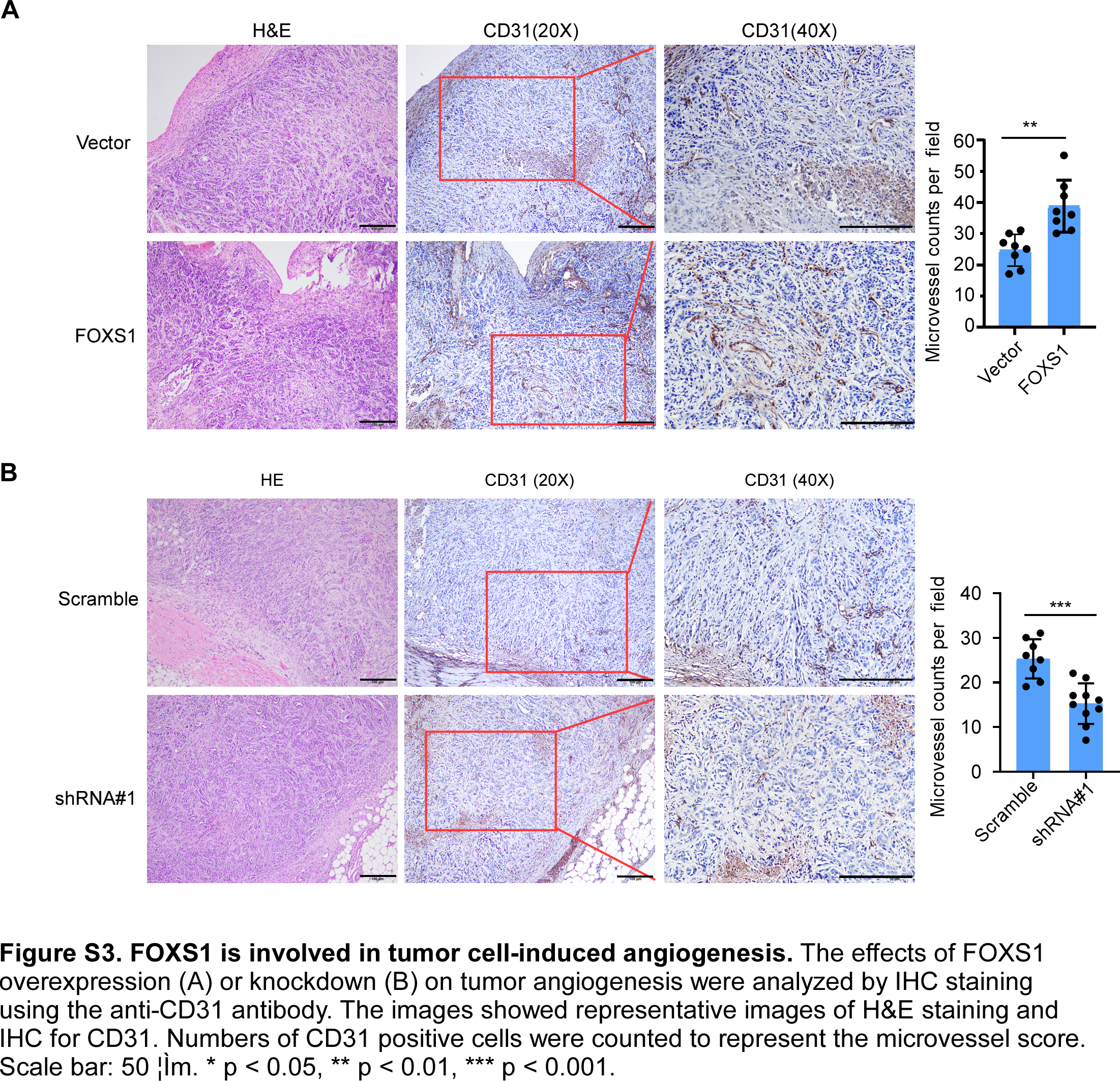

Supplement: Supplementary file 3 [file Image_3.tif]

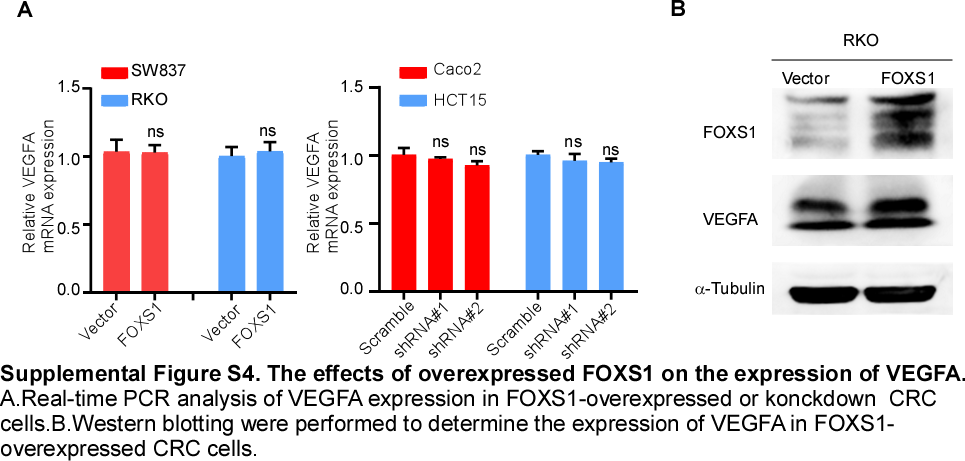

Supplement: Supplementary file 4 [file Image_4.tif]

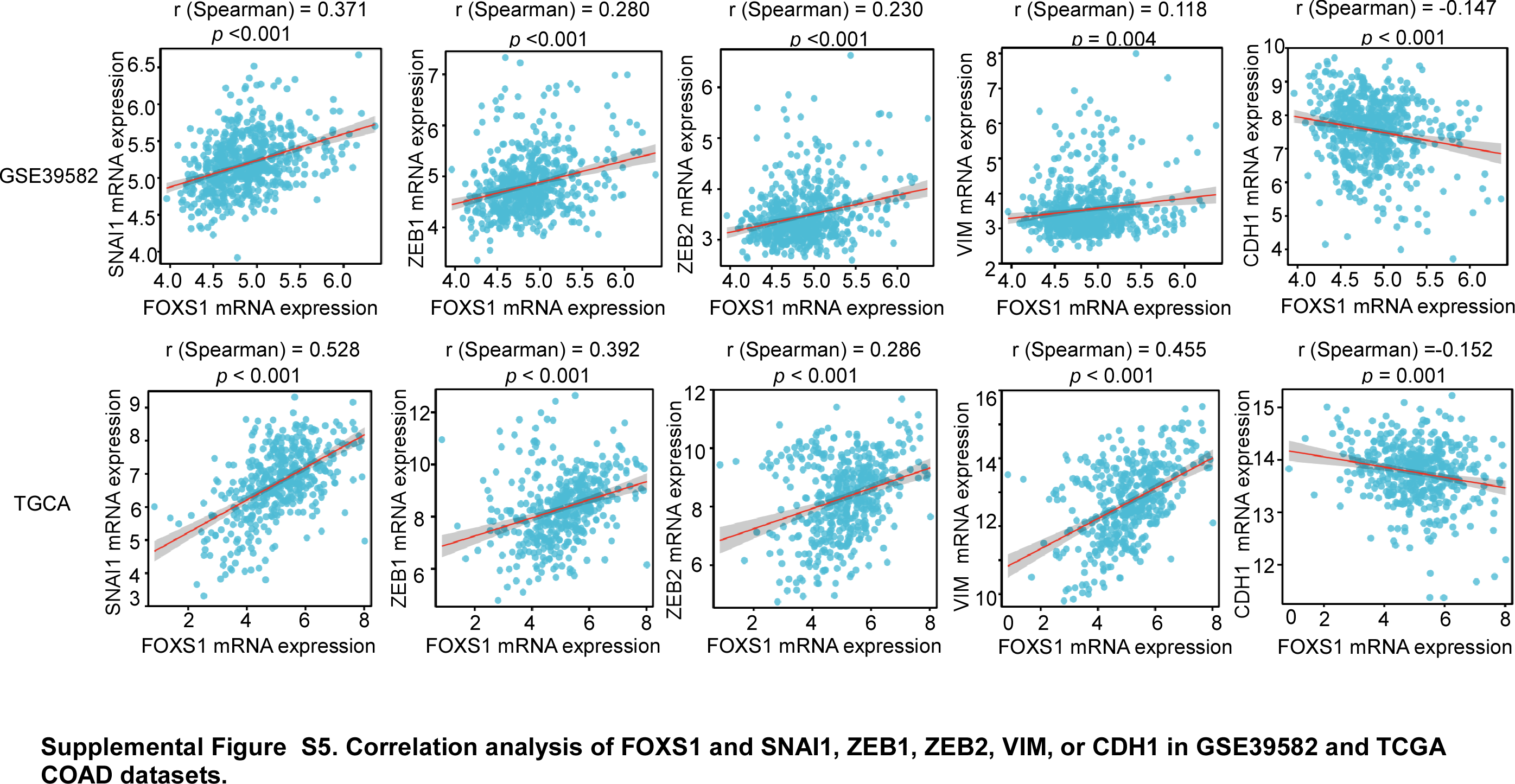

Supplement: Supplementary file 5 [file Image_5.tif]
